# Comparative Mitochondrial Genomic and Phylogenetic Study of Eight Species of the Family Lonchodidae (Phasmatodea: Euphasmatodea)

**DOI:** 10.3390/genes16050565

**Published:** 2025-05-10

**Authors:** Ting Luo, Qianwen Zhang, Siyu Pang, Yanting Qin, Bin Zhang, Xun Bian

**Affiliations:** 1 Key Laboratory of Ecology of Rare and Endangered Species and Environmental Protection (Guangxi Normal University), Ministry of Education, Guilin 541006, China; luot2024@163.com (T.L.); zhangqianwen@163.com (Q.Z.); pangsiyu0820@outlook.com (S.P.); qinyanting2019@163.com (Y.Q.); 2 College of Life Science, Guangxi Normal University, Guilin 541006, China; 3 College of Life Sciences & Technology, Inner Mongolia Normal University, Hohhot 010022, China

**Keywords:** stick insects, mitogenome, phylogenetic analysis

## Abstract

**Background:** Lonchodidae is the largest family within the order Phasmatodea, and although many studies have been conducted on this family, the monophyly of the family has not been established. **Methods:** Eight mitogenomes from Lonchodidae, including the first complete mitogenomes of four genera, were sequenced and annotated to explore their features and phylogenetic relationships. **Results:** The total length ranged from 15,942–18,021 bp, and the mitogenome consisted of 13 protein-coding genes (PCGs), 22 tRNA genes, 2 rRNA genes, and a control region (CR). *atp8* had the highest A + T content in Lonchodidae, except for *Neohirasea stephanus* and *Asceles clavatus*, in which the highest A + T contents were detected in *nad6*. The phylogenetic trees were reconstructed via Bayesian inference (BI) and maximum likelihood (ML) based on the PCG123 and PCG12 datasets. As the phylogenetic trees show, Necrosciinae is recognized as monophyletic, but the monophyly of Lonchodinae has not been supported. Gene deletion and rearrangement have occurred mainly in Lonchodidae and Aschiphasmatidae. The most common reason for gene rearrangements was tandem duplication random loss (TDRL), but *trnI* of *Stheneboea repudiosa* inverted into the CR. In addition, genes within the same family or genus share related sequences and conserved gene blocks. **Conclusions:** we expanded the mitochondrial genomic data for this family, thereby establishing a foundational dataset for future studies.

## Simple Summary

The Lonchodidae family is subject to phylogenetic uncertainties. In this study, the mitochondrial genomes (mitogenomes) of eight species belonging to two subfamilies of Lonchodidae were sequenced and annotated to explore their inter- and intrafamily phylogenetic relationships. A comparison of all available well-characterized mitogenomes revealed that Phasmatodea shares four types of gene rearrangements.

## 1. Introduction

Phasmatodea exhibits stunning camouflage abilities by mimicking various plant structures, serving as a classic model for investigating the adaptive evolution of insects [1,2,3,4]. Phasmatodea has a moderate level of species diversity, with more than 3500 valid species in the world assigned to 14 families, which are distributed mainly in the tropics and subtropics [5,6]. Lonchodidae, the most species-rich family in Phasmatodea, comprises 1243 valid species [5,7]. However, the morphological conservatism exhibited among Phasmatodea species poses significant challenges for classification systems relying on anatomical characteristics, necessitating molecular data to help with species identification and reconstruct a phylogenetic tree within Phasmatodea [8,9,10].

Insect mitochondria are semiautonomous eukaryotic organelles with fully functional gene expression machinery [11]. Mitogenomes have the advantages of highly conserved gene content, rapid evolution and maternal inheritance, all of which make them useful as molecular markers for phylogenetic analysis [12,13,14,15]. While the reliability of mitogenomes as phylogenetic markers remains debated, comparative mitogenomic analyses across species can elucidate their phylogenetic relationships and evolutionary affinities [16,17,18]. Insect mitogenomes generally encode 37 genes, including 13 protein-coding genes (PCGs), 22 tRNA genes (tRNAs), 2 rRNA genes (*rrnS* and *rrnL*), and an A + T-rich region [12,19,20]. Gene rearrangements include transposition, inversion, and inverse transposition [20,21] and serve as valuable markers for conducting thorough phylogenetic studies in certain lineages [22,23]. Tandem duplication random loss (TDRL) is a major gene rearrangement operation in insect mitogenomes, such as those of Ephemeroptera [24], Hemiptera [25,26], and Thysanoptera [27]. Four types of rearrangements have been identified in Phasmatodea [2,28,29,30].

Although mitogenomes have shown potential in insect systematics studies, the study of mitogenomes in Lonchodidae is limited. Currently, the NCBI database contains 32 complete mitogenome sequences of stick insects [31].

Lonchodidae is divided into two subfamilies, Necrosciinae and Lonchodinae, which are frequently revised. The phylogenetic relationships of these species remain a topic of ongoing debate [4,32]. Xu et al. supported Lonchodidae as a polyphyletic group in which Lonchodinae was divided into two clades [33], as was also the case in other studies [2,30,34]. However, Yuan et al. restored Lonchodinae and Necrosciinae as monophyletic groups, but Lonchodidae was still polyphyletic [28], as reported by Chen et al. [7]. In recent years, there has been a surge in the reporting of new species in Lonchodidae [35,36,37,38,39,40,41], and some genera in the family have been continually revised based on morphological data or morphology combined with molecular data [42,43,44,45]. However, the phylogenetic relationships between internal groups in Lonchodidae have not been well resolved [7,46]. More adequate molecular data on Lonchodinae, including mitogenomes, that can elucidate the phylogenetic distribution and evolutionary origin of its gene rearrangements are needed.

In this study, we sequenced and annotated eight complete mitogenomes from the Lonchodidae family, including the first reported mitogenomes for four distinct genera. Determining the mitochondrial gene order, base composition, and gene rearrangements within Lonchodidae can help elucidate its phylogeny. Furthermore, forty-seven mitogenomes were used to construct phylogenetic relationships to infer the relationships of Lonchodidae.

## 2. Materials and Methods

### 2.1. Taxon Sampling and Sequencing

In this study, eight adult individuals of Lonchodinae were collected from the Guangxi and Yunnan, China. The voucher specimens were stored in absolute ethanol at −4 °C in the College of Life Sciences, Guangxi Normal University. Total genomic DNA was extracted from the hind leg of each adult sample via a TIANamp Genomic DNA Kit (TIANGEN, Beijing, China) and then sequenced using 150 bp PE on the Illumina NovaSeq platform (Berry Genomics, Beijing, China).

### 2.2. Genome Annotation and Sequence Analysis

Eight sequences have been uploaded to NCBI (Table S1), and the base content distribution and mass distribution of the sequenced are shown in Figures S1 and S2. The raw paired-end reads were filtered to obtain high-quality clean reads via CLC Genomics Workbench 12 (CLC Bio, Aarhus, Denmark) with default parameters [47]. The mitogenomes were subsequently assembled via NOVOPlasty v.4.2.1 [48]. The MITOS2 web server, which is based on the Galaxy platform (https://usegalaxy.eu/root?tool_id=toolshed.g2.bx.psu.edu%2Frepos%2Fiuc%2Fmitos2%2Fmitos2%2F2.1.3%20galaxy0, accessed on 28 September 2024) was used for preliminary annotation [49] and manually checked by MEGA v.11 [50]. The relative synonymous codon usage (RSCU) value was analysed [51]. A comparative circular genome map was drawn with the BLAST Ring Image Generator [52].

### 2.3. Phylogenetic Analysis

Forty-seven stick insect mitogenomes, including 8 newly obtained sequences in this study and 39 additional sequences retrieved from NCBI (Table S2), were used to investigate the evolutionary status of Lonchodidae. Embioptera (*Eosembia* sp. FS-2017 KX091852 [31]) and Orthoptera (*Sericgryllacris xiai* Liu and Zhang, 2001 KX057734 [53] and *Homogryllacris anelytra* Shi, Guo and Bian, 2012 KX057738 [53]) were selected as the outgroups. Mitogenomes were aligned in batches using MAFFT v7.505 [54] with the auto strategy and codon-based alignment mode. The aligned sequences were concatenated into four sequence matrices: (a) the PCG matrix with 7,582 bp, corresponding to the first and second codon positions of PCGs (PCG12); (b) the PCG matrix with 11,373 bp, corresponding to all codon positions of the PCGs (PCG123); (c) the PCGRNA matrix with 10,079 bp, corresponding to the first and second codon positions of PCGs and 2 rRNAs (PCG12 + 2R); and (d) the PCGRNA matrix with 13,869 bp, corresponding to all codon positions of the PCGs and 2 rRNAs (PCG123 + 2R). The analysis of sequence divergence heterogeneity within the datasets was conducted using AliGROOVE with the default sliding window size [55]. These pairwise distances were evaluated against the distance distribution derived from the entire dataset. The resulting metric ranges from −1 to +1: A value of −1 indicates that the focal group exhibits distances to external lineages that deviate from the dataset-wide average, whereas +1 reflects distances that match the average. In AliGROOVE matrices, darker blue hues in the color-coded similarity scores reflect stronger non-random congruence between pairwise sequence alignments [55]. The low heterogeneity suggests the suitability of these datasets for phylogenetic analyses [56]. The PCG12 and PCG123 matrices were the most suitable for phylogenetic tree reconstruction (Figure S3), because they exhibited deeper blue similarity scores with no significant difference between sequences.

ModelFinder v2.2.0 [57] was used to select the best-fit partition model (Edge-linked) using Bayesian information criterion (BIC criterion) (Table S4). Bayesian inference (BI) analysis was used for phylogenetic reconstruction with MrBayes 3.2.7 [58] with PCG analyzed under the site-homogeneous model (GTR + F + I + G4) (Table S4) and amino acid (AA) sequences under the mtREV + F+I + G4 model (Table S4) for phylogenetic reconstruction. The analysis was conducted with two Markov chain Monte Carlo (MCMC) runs, each with four chains (three heated and one cold), run for 2,000,000 generations, with tree sampling every 1000 generations and a burn-in of 25% [59]. If the BI tree results show that the average standard deviation of split frequencies (ASDSF) is less than 0.01, the BI operation is considered to have converged [60]. Maximum likelihood (ML) analysis was performed using IQ-TREE v2.2.0 [61] for phylogenetic reconstruction: PCG was analyzed under the GTR + F+I + G4 model (Table S4), and AA sequences were partitioned with separate models (Table S4), with 5000 standard bootstrap repetitions for tree support [60]. Finally, the phylogenetic tree was built in Interactive Tree Of Life (iTOL) (https://itol.embl.de/, accessed on 2 February 2025) [62].

## 3. Results and Discussion

### 3.1. Mitochondrial Genomic Characterization of Eight Species

**Figure 1:**
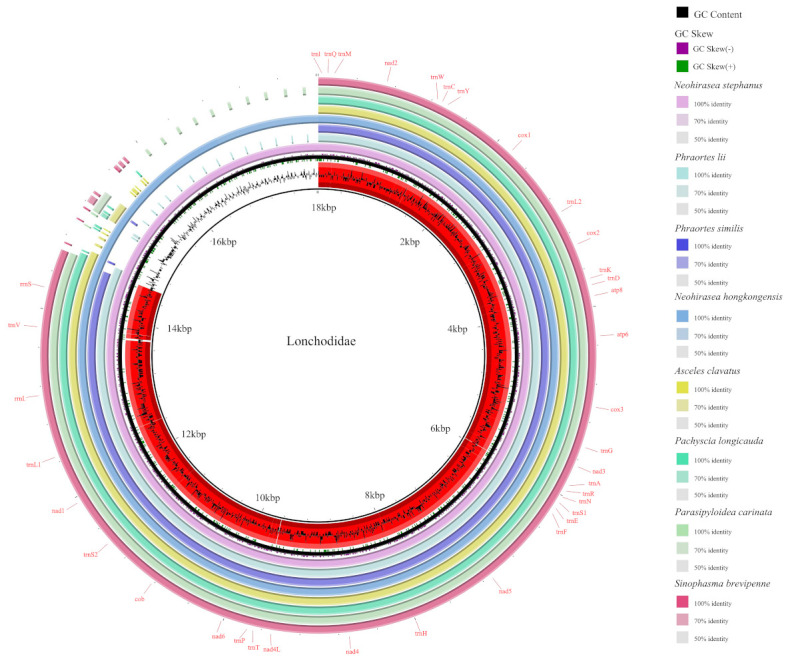
Comparative genomic circle map of eight newly sequenced species. Note: The innermost layer is the GC content, the second layer is the GC skew, and the third layer is the self-proportional sequence of the reference genome (*N. stephanus*). The eight outer layers represent the structure aligned to the reference sequence, which contains *Phraortes lii*, *Phraortes similis*, *Neohirasea hongkongensis*, *A. clavatus*, *Pachyscia longicauda*, *Parasipyloidea carinata*, and *Sinophasma brevipenne*. Thirty-seven genes were located in the outermost layers. This comparative genomic circle map suggests that the eight species have different genome lengths.

The eight newly completed mitogenomes were consistently circular in structure, ranging from 15,942–18,021 bp in size. The genomes presented an average GC content of 22.3%. The mitogenomes comprised a total of 37 genes, including 13 PCGs, 22 tRNAs, 2 rRNAs, and a control region (CR) (Figure 1). There were 23 out of 37 genes (9 PCGs and 14 tRNAs) being encoded on the majority strand (J-strand), while the remaining 14 genes (4 of the 13 PCGs, 8 tRNAs, and 2 rRNAs) were located on the minority strand (N-strand).

### 3.2. Codon Usage

**Figure 2:**
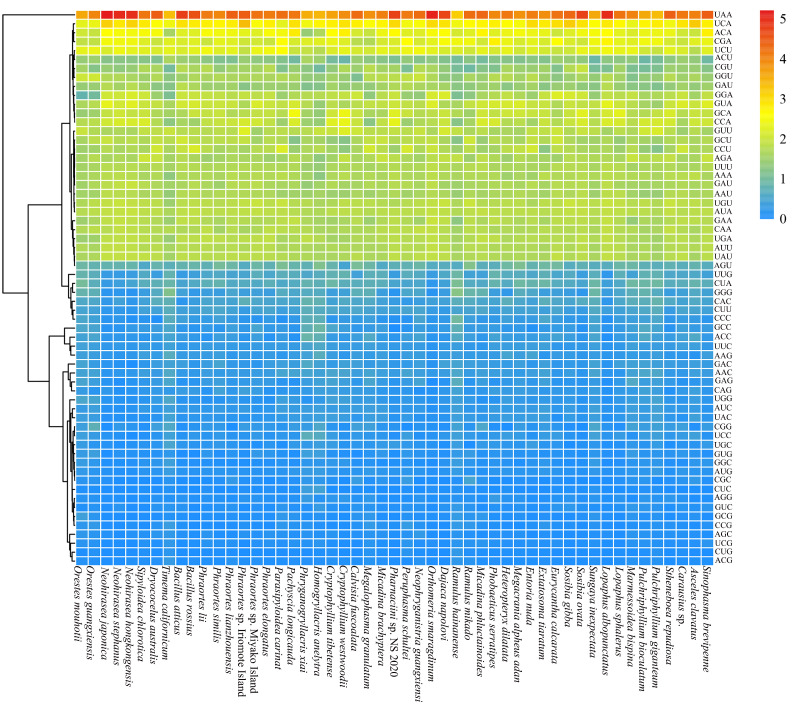
Heatmap of the RSCU of the mitogenomes of 47 stick insects.

The majority of PCGs began with ATN, with two notable exceptions: *P. longicauda* utilizes TTG (as observed in *Caligula boisduvalii* [63]), and *N*. *hongkongensis* employs GTG (paralleling the pattern in *Coridius chinensis* [64]). Most PCGs terminated with the codon TAA, while some ended with TAG or incomplete termination codons TA or T (Table S3). The third transcription of TA or T could add a poly(A) sequence to explain the absence of a termination codon [65], which has been observed in other insects [66,67,68]. The mitogenomes of the 47 stick insects exhibited a strong bias towards UUA, followed by UCA (Figure 2).

### 3.3. AT Bias

**Figure 3:**
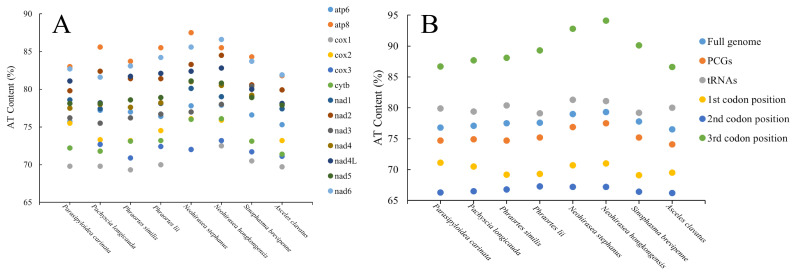
The A + T contents of (**A**) the 13 PCGs in eight Phasmatodea species and (**B**) transfer RNAs (tRNAs); the whole, first, second, and third positions of PCGs; the CR, and the full mitogenome in the heavy strand.

The PCGs presented high A + T contents ranging from 69.3–87.5%. The A + T content at the third codon position (86.6–94.1%) was much greater than that at the other codon positions (66.3–71.1%). Among all the PCGs, except for *nad6* in *N*. *hongkongensis* and *A. clavatus*, the highest A + T content was exhibited in *atp8*, whereas *cox1* presented the lowest A + T content (Figure 3).

### 3.4. Genetic Rearrangement

**Figure 4:**
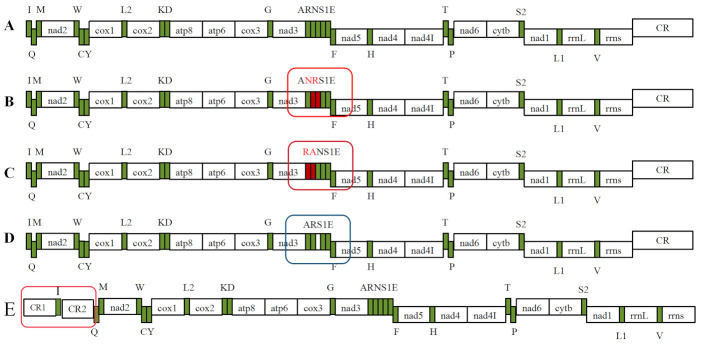
Diagram of the gene rearrangement patterns of the mitogenome of stick insects. (**A**) Original gene sequence alignment order of Phasmatodea; (**B**) *O. smaragdinum*, *D. napolovi*; (**C**) *Carausius* sp.; *M. granulatum*; (**D**) *M. brachyptera*; (**E**) *S. repudiosa*. Notes: the red box indicates the presence of genetic rearrangement types, and the blue box indicates genetic deletion.

Mitochondrial sequences within invertebrate lineages are conserved, and fewer rearrangements occur [69]. We compared the arrangement orders of 37 genes and the CRs in the mitogenomes of Phasmatodea insects and identified four arrangement patterns (Figure 4) [2,28,29,30]. The gene arrangement order of the mitogenomes of the eight Lonchodidae species obtained in this study was consistent with that of the ancestral insect mitogenomes (Figure 4A) [28]. *Orthomeria smaragdinum* and *Dajaca napolovi* presented a gene block rearrangement of *trnR*–*trnN* to *trnN*–*trnR* (Figure 4B). *Carausius* sp. and *Megalophasma granulatum* exhibited a reordering type from *trnA*–*trnR* to *trnR*–*trnA* (Figure 4C). The *trnN* gene of *Micadina brachyptera* is lacking, resulting in the sequence *trnA*–*trnR*–*trnS1*–*trnE* (Figure 4D). In *S. repudiosa*, the *trnI* gene was inverted and transferred to the CR (Figure 4E).

Rearrangement of the PCGs in Phasmatodea occurs in the families Lonchodidae and Aschiphasmatidae. In Aschiphasmatidae, only the gene block *trnR*–*trnN* was rearranged to *trnN*–*trnR*, whereas Lonchodidae contains the remaining types. The rearrangements of *O. smaragdinum* and *D. napolovi* were identified as a plesiomorphic feature of Aschiphasmatidae, and TDRL was used to explain this phenomenon, in which *trnR*–*trnN* was duplicated as *trnR*–*trnN*–*trnR*–*trnN* and then randomly lost as *trnN*–*trnR*. *Carausius* sp. and *M. granulatum* also seemed to have undergone TDRL, in which *trnA*–*trnR* was first duplicated as *trnA–trnR–trnA–trnR*, the first *trnA* was lost, the last *trnR* was subsequently lost, and the gene block ultimately become *trnR–trnA*. However, *M. granulatum* also reexhibited the transposition of ancestral insect arrangements [30]. The reason for *M. brachyptera* genetic rearrangements could also be TDRL, which was the generally accepted hypothetical mechanism for gene rearrangement [20,70,71]. Insect mitogenome rearrangements are usually attributed to tandem duplications caused by replication errors, the most common type of which is TDRL [23]. This could be explained by gene blocks resulting from successive rounds of tandem replication of consecutive gene fragments. To maintain the normal function of the mitotic genome, one of the duplicated gene blocks randomly loses its function and becomes a pseudogene to be further selected, which can even be lost altogether in subsequent evolutionary events [72,73]. *trnI* inverted into the CR in *S. repudiosa* because of internal translocation. Owing to the low homology of CR1 and CR2, which cannot be translated, CR1 lacked a repeat series, but CR2 contained a tandem repeat series. It was hypothesized that *trnI* was inverted first from the positive strand to the negative strand and then randomly inserted into the middle of the CR [28]. Genetic rearrangement leads to the existence of two control regions in Hymenoptera (*Aphidius gifuensis*) [74].

### 3.5. Phylogenetic Relationships

**Figure 5:**
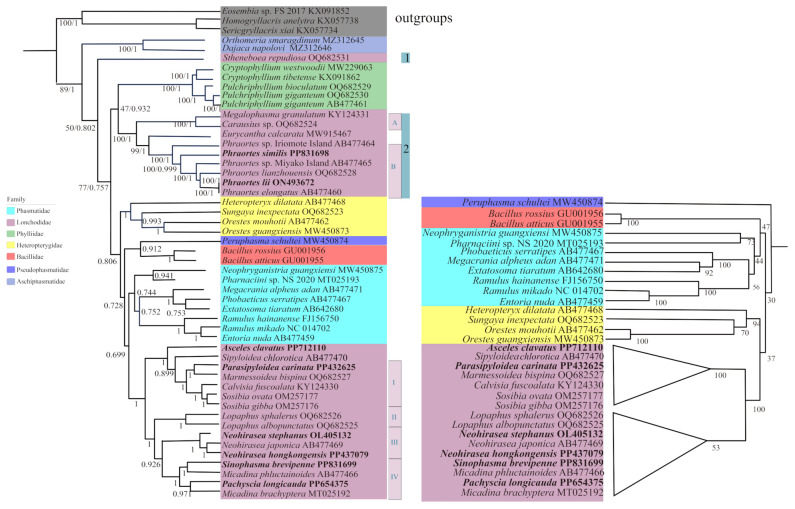
Phylogenetic tree obtained from BI and ML analysis based on PCG123, with the numbers on the branches indicating bootstrap percentages from ML (**left**) and posterior probabilities as determined from BI (**right**). Note: Labeled triangles indicate that BI and ML have the same topology.

**Figure 6:**
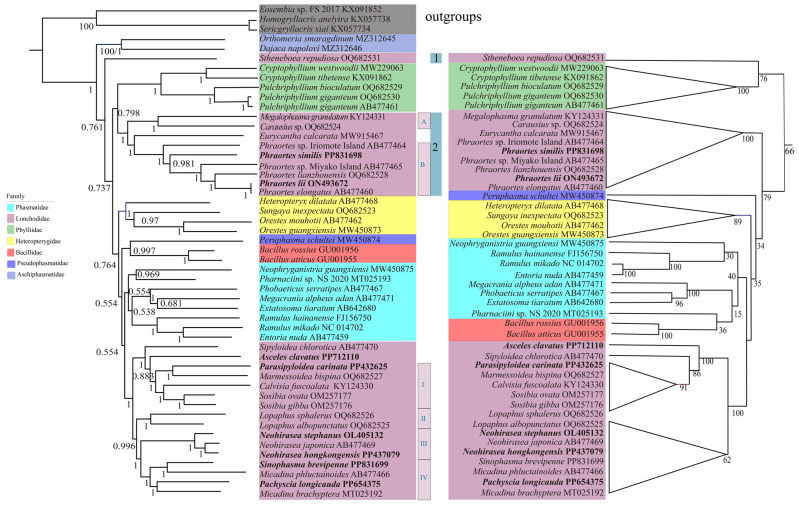
Construction of BI and ML phylogenetic tree analysis of the nucleotide dataset based on PCG12, with the numbers on the branches indicating bootstrap percentages from ML (**left**) and posterior probabilities as determined from BI (**right**). Note: Labeled triangles indicate that BI and ML have the same topology.

This study focuses on the phylogenetic tree constructed for PCG123 in the main text (Figure 5), and the tree constructed based on the PCG12 dataset is shown in Figure 6. Branches with Bayesian posterior probability (PP) < 0.85 or ML bootstrap (BS) < 50 are considered not well supported, indicating that the sister group relationships in the ML or BI trees are not robust [75].

The results from the ML and BI tree analyses support the following relationships: (a) Aschiphasmatidae is identified as a sister group to the other families within Neophasmatodea, which is consistent with the results of other studies [10,28,30]; (b) Phasmatidae is a monophyletic group in the ML and BI trees [7], contrasting with Song et al. [2], who established Phasmatidae as polyphyletic; (c) Lonchodidae is a polyphyletic group [4,28,76]; (d) the Heteropterygidae family is divided into three subfamilies [28,33,77], in which our results support ((Dataminae + Obriminae) + Heteropteryginae) [28]; and (e) Pseudophasmatidae is an independent clade, which is consistent with the findings of a previous study [28].

Our findings did not support the monophyly of Lonchodidae, and sister relationships of Necrosciinae and Lonchodinae were not identified. Necrosciinae was well clustered and monophyletic, but Lonchodinae was a polyphyletic group that could be divided into two clades (clade 1 contains *S. repudiosa* OQ682531 [28], and clade 2 includes all remaining species of the subfamily). Amino acid-based phylogenetic trees in Figures S4 and S5. The results align with those constructed using nucleotide sequences, in which the monophyly of Lonchodinae is not supported, while the monophyly of Necrosciinae is recovered. These results are consistent with the findings of Xu et al. [33]. However, Yuan et al. reported that Lonchodinae and Necrosciinae are monophyletic [28]. In clade 2, branch A included (*M. granulatum* + *Carausius* sp.); branch B consisted of six species of *Phraortes,* of which *Phraortes* sp. Iriomote Island was a sister group with (*P*. *similis* + (*Phraortes* sp. Miyako Island + (*P*. *lianzhouensis* + (*P*. *lii* + *P*. *elongatus*)))). Clade 2 was (branch A + (*Eurycantha calcarata +* branch B)) in both the ML and BI trees. In Necrosciinae, branch I contained (*P. carinata + Marmessoidea bispina*) + (*Calvisia fuscoalata +* (*Sosibia ovata + Sosibia gibba*)). Branch II consisted of two species of the *Lopaphus* genus (*Lopaphus sphalerus* and *Lopaphus albopunctatus*), in which they were in a sister group, and their monophyletic group was recovered. Branch III included *N. stephanus + N*. *japonica* + *N*. *hongkongensis* and established the monophyletic group of the genus. (*S. brevipenne + Micadina phluctainoides*) + (*P. longicauda* + *M. brachyptera*) constituted branch IV, but the monophyly of *Micadina* could not be supported. The BI and ML trees of PCG123 and the ML tree of PCG12 supported (*A. clavatus +* (*Sipyloidea chlorotica +* branch I)) + (branch II + (branch III + branch IV)), whereas (*S. chlorotica + (A. clavatus* + branch I)) + (branch II + (branch III + branch IV)) was supported in the BI tree of PCG12.

In Phasmatodea, some differences were detected between the ML and BI trees. The results of the BI analysis at the PCG123 and PCG12 are consistent, and their phylogenetic relationships were clade 1 + ((Phylliidae + clade 2) + (Heteropterygidae + ((Pseudophasmatidae + Bacillidae) + (Phasmatidae + Necrosciinae)))). In the ML tree of PCG12, it supported a sister–group relationship (clade 1 + Phylliidae) + (clade 2 + (Pseudophasmatidae + (Heteropterygidae + ((Phasmatidae + Bacillidae) + Necrosciinae)))). In the ML tree of PCG123, clade 1 was a sister group with ((Phylliidae + clade 2) + (Pseudophasmatidae + ((Bacillidae + Phasmatidae) + (Heteropterygidae + Necrosciinae)))).

### 3.6. Relationships Between Gene Rearrangement and Phylogeny

Gene deletions and rearrangements occurred mainly in Lonchodidae and Aschiphasmatidae. All the genomes of all the species are circular [28,30], except *M. granulatum* and *M. brachyptera*, whose genome is linear [2,29]. Notably, the most closely related *Carausius* sp. and *M. granulatum* presented translocations of the *trnA* and *trnR* genes. Both *O. smaragdinum* and *D. napolovi* of the Aschiphasmatidae family had *trnR*–*trnN* rearranged to *trnN*–*trnR.* This may indicate a similar profile of gene alterations in closely related species in which the types of gene rearrangements and the gene order have a certain degree of randomness across clades. A unique gene order is conserved within the same family or genus [72]. As comparative genomic features, gene rearrangements are more conserved in most taxa and occur less frequently, whereas the results of the present study revealed that species from the same family or close relatives have similar gene orders, gene losses, and more conserved gene blocks, which may provide important information for phylogenetic relationships [78,79,80]. Due to the conservation of the mitogenome order and the rearrangement in closely related species, the rearrangement is hypothesized to have existed in the ancestral mitochondrial genome as well. However, the discovery of more species with genetic rearrangements and a discussion of their affinities are needed to clarify that closely related species may have the same type of rearrangement.

## 4. Conclusions

To elucidate the mitogenomic structure of Lonchodidae and understand inter- and intrafamily phylogenetic relationships, eight mitochondrial genomes were sequenced, which included the first complete mitogenomes of four genera. Four gene rearrangements, including inversions and transversions, that occurred in Lonchodidae and Aschiphasmatidae were identified. Related gene orders and rearrangements are more likely to occur within the same family. Based on 13 PCG datasets, a phylogenetic relationship for Phasmatodea was obtained in which Lonchodidae is a polyphyletic group and Necrosciinae is monophyletic. Stable intra-Lonchodidae polyphyletic relationships and the monophyly of *Lopaphus* and *Neohirasea* are supported. Adding new mitochondrial genomic data and summarizing the types of rearrangements present could enhance our understanding of Phasmatodea mitogenomes, thereby aiding in the resolution of their phylogenetic relationships.

## Data Availability

The newly sequenced eight mitogenome sequences have been submitted at NCBI (Acc. number OL405132, PP437079, PP712110, PP654375, PP432625, ON493672, PP831698, PP831699).

## Supplementary Materials

The following supporting information can be downloaded at: https://www.mdpi.com/article/10.3390/genes16050565/s1, Figure S1: Sequencing base content distribution map for eight species. Figure S2: Mass distribution of sequenced base in eight species. Figure S3: Heterogeneity test for different datasets. Figure S4: Construction of ML phylogenetic tree analysis of amino acid dataset based on PCG123. Figure S5: Construction of BI phylogenetic tree analysis of amino acid dataset based on PCG123. Table S1: The mitochondrial genome information and raw data for eight species were obtained in this study. Table S2: List of samples included in phylogenetic analysis. Table S3: Start and termination codons of the PCG of eight Lonchodidae species. Table S4: Best-fitting models selected of mitochondrial genomes.
