# Supplementary material for: Comparative Mitochondrial Genomic and Phylogenetic Study of Eight Species of the Family Lonchodidae (Phasmatodea: Euphasmatodea)
Source: Genes (Basel). 2025 May 10;16(5):565. doi: 10.3390/genes16050565 (PMC12111144; doi:10.3390/genes16050565)
Supplement: Supplementary file 1 [file genes-16-00565-s001.zip › Table S4. Best-fitting models selected of mitochondrial genomes.pdf]

Tabel S 4.1 Based on PCG123, partition strategies used in phylogenetic analyses in ML tree.

Subset	Subset Partitions	Best-fitting models in ML
1	atp6_mafft+nad2_mafft	GTR+F+I+R5
2	atp8_mafft	TN+F+I+G4
3	cox1_mafft+cox2_mafft+cytb_mafft+nad3_mafft	GTR+F+I+G4
4	cox3_mafft	GTR+F+I+G4
5	nad1_mafft+nad4L_mafft+nad4_mafft	GTR+F+I+R5
6	nad5_mafft	TIM+F+I+R5
7	nad6_mafft	TN+F+I+G4

Tabel S 4.2 Based on PCG12, partition strategies used in phylogenetic analyses in ML tree

Subset	Subset Partitions	Best-fitting models in ML
1	atp6_mafft+nad2_mafft	GTR+F+I+R5
2	atp8_mafft	TN+F+I+G4
3	cox1_mafft+cox2_mafft	GTR+F+I+G4
4	cox3_mafft	GTR+F+I+G4
5	cytb_mafft+nad3_mafft	GTR+F+I+G4
6	nad1_mafft+nad4L_mafft+nad4_mafft	GTR+F+I+R5
7	nad5_mafft	TIM+F+I+R5
8	nad6_mafft	TN+F+I+G4

Tabel S 4.3 partition strategies used in phylogenetic analyses PCGs in BI tree.

Subset	Subset Partitions	Best-fitting models in BI
1	atp6_mafft+nad2_mafft	GTR+F+I+G4
2	atp8_mafft	GTR+F+I+G4
3	cox1_mafft+cox2_mafft+cytb_mafft+nad3_mafft	GTR+F+I+G4
4	cox3_mafft	GTR+F+I+G4
5	nad1_mafft+nad4_mafft	GTR+F+I+G4
6	nad4L_mafft+nad5_mafft	GTR+F+I+G4
7	nad6_mafft	GTR+F+I+G4

Tabel S 4.4 partition strategies used in phylogenetic analyses AA in ML tree.

Subset	Subset Partitions	Best-fitting models in BI
1	atp6_mafft	mtART+F+I+G4
2	atp8_mafft	Q.plant+F+G4
3	cox1_mafft	mtZOA+F+I+G4
4	cox2_mafft_cox3_mafft_cytb_mafft_nad3_mafft	mtART+F+I+R5
5	nad1_mafft	mtZOA+F+G4
6	nad2_mafft_nad6_mafft	mtMet+F+R5
7	nad4L_mafft	mtInv+G4
8	nad4_mafft_nad5_mafft	mtInv+F+I+R5

Tabel S 4.5 partition strategies used in phylogenetic analyses AA in BI tree.

Subset	Subset Partitions	Best-fitting models in BI
1	atp6_mafft	mtREV+F+I+G4
2	atp8_mafft	mtREV+F+I+G4
3	cox1_mafft	mtREV+F+I+G4
4	cox2_mafft_cox3_mafft_cytb_mafft_nad3_mafft	mtREV+F+I+G4
5	nad1_mafft_nad4L_mafft	mtREV+F+I+G4
6	nad2_mafft_nad6_mafft	mtREV+F+I+G4
7	nad4_mafft_nad5_mafft	JTT+F+I+G4
