# Supplementary figures and images for: Comparative Mitochondrial Genomic and Phylogenetic Study of Eight Species of the Family Lonchodidae (Phasmatodea: Euphasmatodea)
Source: Genes (Basel). 2025 May 10;16(5):565. doi: 10.3390/genes16050565 (PMC12111144; doi:10.3390/genes16050565)

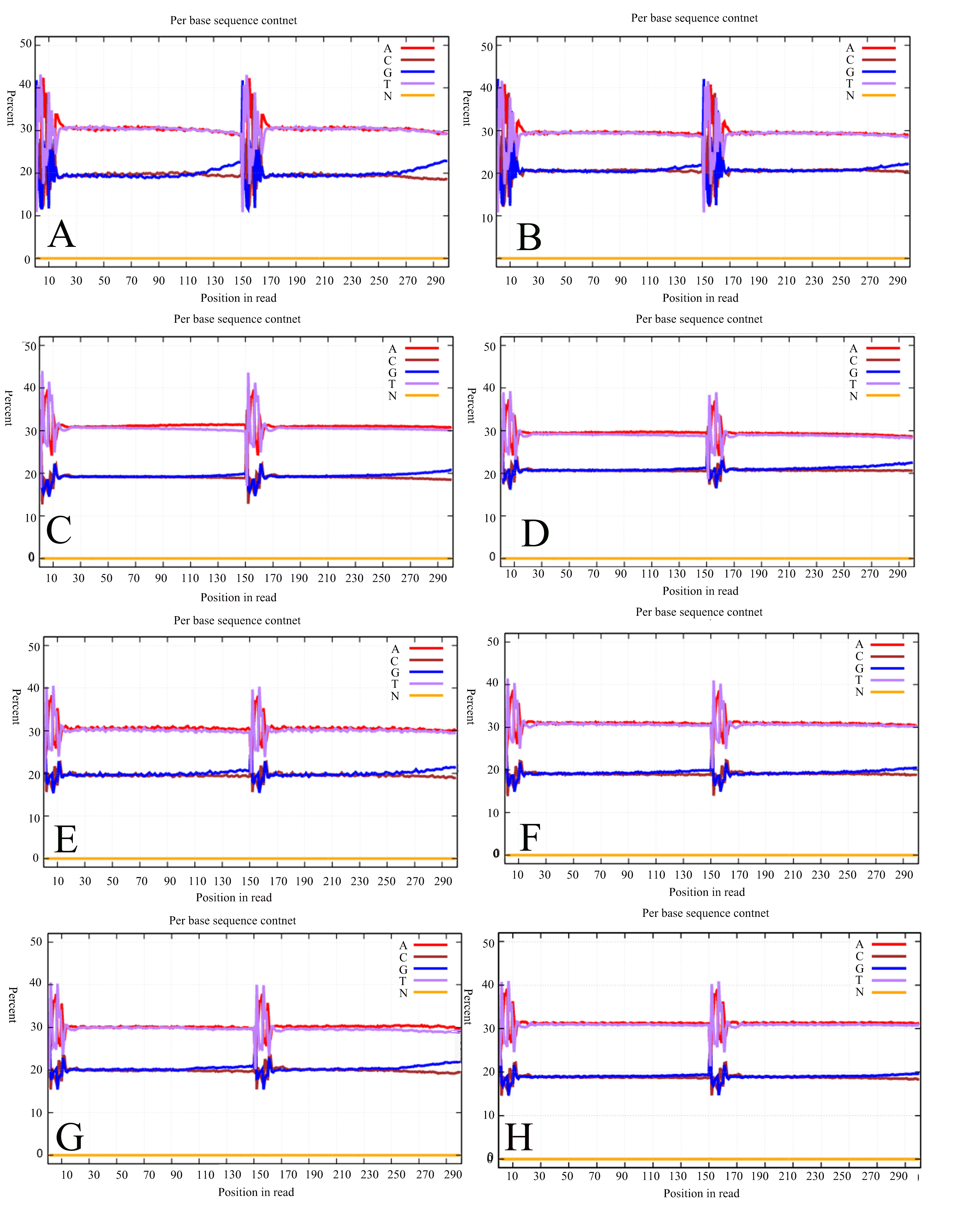

Supplement: Supplementary file 1 [file genes-16-00565-s001.zip › Figure S1. Sequencing base content distribution map for eight species_.png]

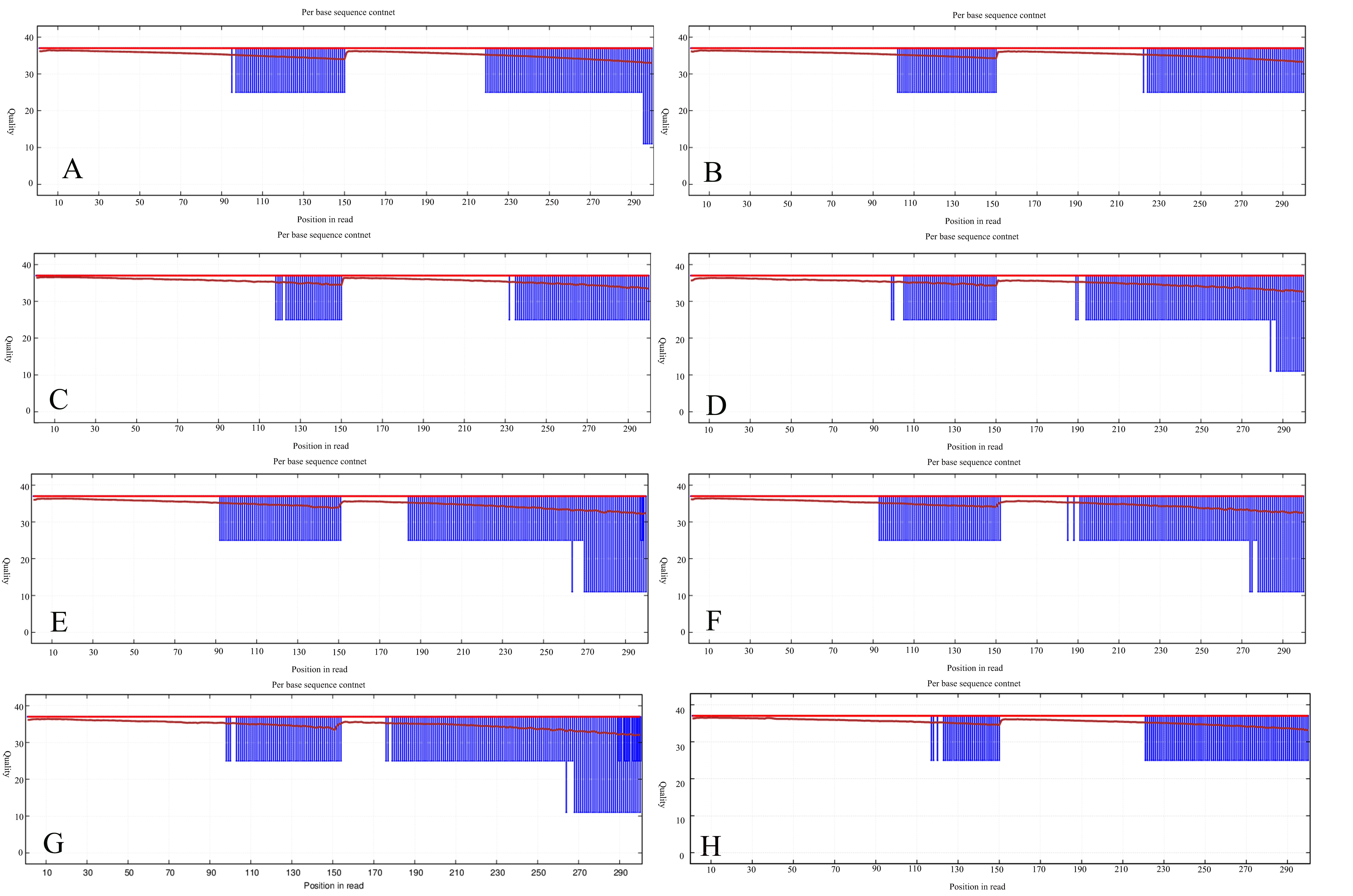

Supplement: Supplementary file 1 [file genes-16-00565-s001.zip › Figure S2. Mass distribution of sequenced base in eight species_.png]
